# Genome Sequence of the Nonpathogenic *Pseudomonas aeruginosa* Strain ATCC 15442

**DOI:** 10.1128/genomeA.00421-14

**Published:** 2014-05-01

**Authors:** Yujiao Wang, Chao Li, Chao Gao, Cuiqing Ma, Ping Xu

**Affiliations:** a State Key Laboratory of Microbial Technology, Shandong University, Jinan, China; b State Key Laboratory of Microbial Metabolism & School of Life Sciences and Biotechnology, Shanghai Jiao Tong University, Shanghai, China

## Abstract

*Pseudomonas aeruginosa* ATCC 15442 is an environmental strain of the *Pseudomonas* genus. Here, we present a 6.77-Mb assembly of its genome sequence. Besides giving insights into characteristics associated with the pathogenicity of *P. aeruginosa*, such as virulence, drug resistance, and biofilm formation, the genome sequence may provide some information related to biotechnological utilization of the strain.

## GENOME ANNOUNCEMENT

*Pseudomonas aeruginosa*, a ubiquitous Gram-negative bacterium, is widespread in nature, inhabiting soil, water, plants, and animals (1, 2). Most strains of this species are opportunistic human pathogens that cause disease in immunocompromised hosts and individuals with cystic fibrosis (2). So far, whole-genome sequences of some *P. aeruginosa* strains, such as *P. aeruginosa* PAO1, *P. aeruginosa* PA7, and *P. aeruginosa* XMG, are publicly available (1–3). Analyses of those genome sequences have provided some useful information about characteristics related to the pathogenicity of *P. aeruginosa*, such as virulence, drug resistance, and biofilm formation (1–3).

Besides the clinical isolates, there are also some environmental *P. aeruginosa* strains. For example, *P. aeruginosa* ATCC 15442 was originally isolated from an animal room water bottle. This strain was neither invasive nor cytotoxic (4). It was thus used as the reference strain in disinfectant testing (5). To better understand the pathogenicity of *P. aeruginosa* and to further improve its biotechnological applications, we sequenced the genome of *P. aeruginosa* ATCC 15442.

The draft genome sequence of *P. aeruginosa* ATCC 15442 was obtained using the Illumina GA system; sequencing was performed by the Chinese National Human Genome Center at Shanghai in China, with a paired-end library. The reads were assembled by using Velvet software (6). Primary coding sequence extraction and initial functional assignment were carried out using the Rapid Annotations using Subsystems Technology (RAST) automated annotation server (7). The G+C content was calculated using the draft genome sequence. The functional description was determined using Clusters of Orthologous Genes (8). The rRNA and tRNA genes were identified by RNAmmer 1.2 (9) and tRNAscan-SE (10), respectively.

The draft genome sequence of *P. aeruginosa* ATCC 15442 has a G+C content of 66.2%. The number of contigs (>100 bp) is 200, and the number of bases is 6,770,586. There are 63 tRNA genes, 11 rRNA operons, and 6,351 putative coding sequences (CDSs) (934 bp average length) in the genome sequence. The coding percentage is 79.6%, and 5,055 CDSs have predicted functions.

There are 573 subsystems represented in the draft genome sequence. In contrast to *P. aeruginosa* PAO1, *P. aeruginosa* PA14, and *P. aeruginosa* XMG, there are no complete pyocyanin production genes in the draft genome sequence. Since pyocyanin produced by *P. aeruginosa* may contribute to infection (11), the absence of complete siderophore production genes might explain the noninvasive and noncytotoxic properties of ATCC 15442. An *lldRPDE* operon is also annotated in the lactate utilization subsystem (12, 13). Biocatalysts containing NAD-independent l-lactate dehydrogenase (encoded by *lldD*) and NAD-independent d-lactate dehydrogenase (encoded by *lldE*) could be used in pyruvate production (14–16), kinetic resolution of 2-hydroxy acid racemic mixtures (17, 18), and 2-oxobutyrate production (19). Therefore, genome scale analysis might be useful for the metabolic engineering of the environmental strain ATCC 15442 to enhance its ability to serve as a useful biocatalyst.

### Nucleotide sequence accession numbers.

This whole-genome shotgun project has been deposited at DDBJ/EMBL/GenBank under the accession no. AYUC00000000. The version described in this paper is the first version, with accession no. AYUC01000000.
